# Surface engineering of earth-abundant Fe catalysts for selective hydrodeoxygenation of phenolics in liquid phase†
†Electronic supplementary information (ESI) available. See DOI: 10.1039/d0sc00983k


**DOI:** 10.1039/d0sc00983k

**Published:** 2020-05-18

**Authors:** Jianghao Zhang, Junming Sun, Libor Kovarik, Mark H. Engelhard, Lei Du, Berlin Sudduth, Houqian Li, Yong Wang

**Affiliations:** a The Gene & Linda Voiland School of Chemical Engineering and Bioengineering , Washington State University , Pullman , WA 99164 , USA . Email: junming.sun@wsu.edu; b Institute for Integrated Catalysis , Environmental Molecular Sciences Laboratory , Pacific Northwest National Laboratory , Richland , WA 99352 , USA . Email: yong.wang@pnnl.gov; c School of Chemistry and Chemical Engineering , Harbin Institute of Technology , Harbin 150001 , China

## Abstract

Development of inexpensive sulfur-free catalysts for selective hydrogenolysis of the C–O bond in phenolics (*i.e.*, selective removal of oxygen without aromatic ring saturation) under liquid-phase conditions is highly challenging. Here, we report an efficient approach to engineer earth-abundant Fe catalysts with a graphene overlayer and alkali metal (*i.e.*, Cs), which produces arenes with 100% selectivity from liquid-phase hydrodeoxygenation (HDO) of phenolics with high durability. In particular, we report that a thin (a few layers) surface graphene overlayer can be engineered on metallic Fe particles (G@Fe) by a controlled surface reaction of a carbonaceous compound, which prevents the iron surface from oxidation by hydroxyls or water produced during HDO reaction. More importantly, further tailoring the surface electronic properties of G@Fe with the addition of cesium, creating a Cs-G@Fe composite catalyst in contrast to a deactivated Cs@Fe one, promotes the selective C–O bond cleavage by inhibiting the tautomerization, a pathway that is very facile under liquid-phase conditions. The current study could open a general approach to rational design of highly efficient catalysts for HDO of phenolics.

## Abstract 2

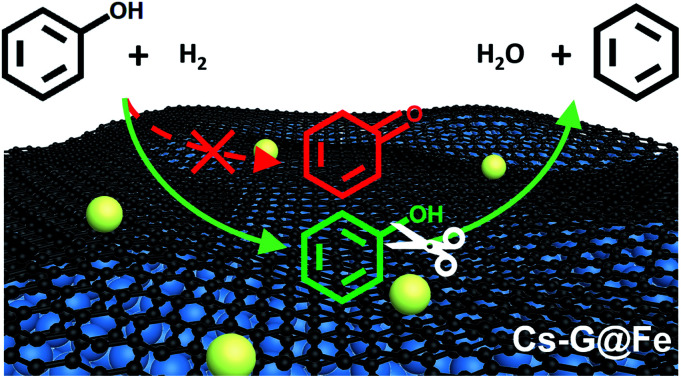
Tailoring the graphene-covered Fe with Cs modifies the surface electronic properties of the catalysts such that selective C–O bond cleavage of phenol is achieved in liquid phase by inhibiting the facile tautomerization followed by ring saturation.

## Introduction

Since its discovery,^1^ 2D graphene has been attracting increased attention due to its potential applications in a variety of areas such as catalysis.^2^ Recently, new surface band structures caused by the electron transfer between metal substrate and graphene overlayers have been demonstrated to affect the adsorption/activation of adsorbed molecules in electrocatalysis.^3^,^4^ Graphene coating has also been reported to be capable of protecting metal surfaces from oxidation.^5^

Hydrodeoxygenation (HDO) plays pivotal roles in biomass conversion, *i.e.*, removing oxygen-containing functional groups from bio-oil. During the HDO of phenolics, selective removal of the oxygen without saturation of the aromatic ring not only minimizes the consumption of valuable hydrogen, but also produces a gasoline blending stock with high octane number, which is critical to the efficient upgrading of bio-oil.^6^ While sulfide-based catalysts have been shown to be promising for selective production of arenes, sulfur contamination is inevitable in the products.^7^ Among the precedent efforts on exploring sulfur-free catalysts,^8^,^9^ several catalysts have been demonstrated to exhibit high selectivity to arenes in gas-phase HDO. However, the ring saturation products were found to be dominant when similar catalysts were used in liquid-phase HDO, a preferred process in biomass conversion which avoids the energy consumption associated with vaporization.^8^,^10^–^13^ We have recently developed a Pd–Fe bimetallic catalyst for selective oxygen removal in gas-phase HDO of phenolics.^9^ Over the Pd–Fe catalyst, oxophilic Fe^14^ is responsible for direct C–O bond cleavage whereas Pd facilitates hydrogen dissociation, improving hydrogen coverage on Fe and facilitating the removal of the hydroxyls generated, which mitigates the oxidation and deactivation of surface Fe.^8^,^15^ When the same Pd–Fe catalyst was applied in liquid-phase reaction conditions, it exhibited low selectivity to arenes due to the facile aromatic ring saturation *via* tautomerization.^16^ To our best knowledge, it is still highly challenging to develop an inexpensive sulfur-free catalyst for selective HDO of phenolics under liquid-phase reaction conditions.^17^

Motivated by the protection of transition metals by graphene overlayer^3^,^5^,^18^ and improved C–O bond hydrogenolysis by alkali metals in homogeneous catalysis,^19^,^20^ herein we report a facile approach by which a heterogeneous and highly stable Fe-based catalyst can be engineered to produce arenes from phenolics with 100% selectivity under liquid-phase conditions. Compared to other alkali metals, Cs has been reported to show higher charge transfer to Fe.^21^ Therefore, Cs was employed in this study to tailor catalyst surface properties. A suite of complementary characterization techniques including scanning transmission electron microscopy (STEM) coupled with electron energy loss spectroscopy (EELS), Raman spectroscopy, temperature programmed surface reaction with H_2_ (H_2_-TPSR), *in situ* X-ray photoelectron spectroscopy (XPS), and *in situ* attenuated total reflectance Fourier-transform infrared spectroscopy (ATR-FTIR) reveal that the thin graphene overlayer prevents the Fe surface from oxidation, making the graphene/Fe composite (G@Fe) a durable Fe-based catalyst resistant to oxidation in HDO of phenol. More importantly, upon modification of this graphene/Fe composite with an alkali metal (*i.e.*, Cs, denoted as Cs-G@Fe), the graphene overlayers could also regulate the Fe–alkali metal interactions. As such, rather than a poison of Fe surface on Cs@Fe, tailored surface electronic properties were observed on the Cs-G@Fe composite, leading to exclusive arene production in HDO of phenolics in liquid phase *via* inhibiting tautomerization and aromatic ring saturation of phenolics.

## Results and discussion

### Structure of the catalysts

**Fig. 1:**
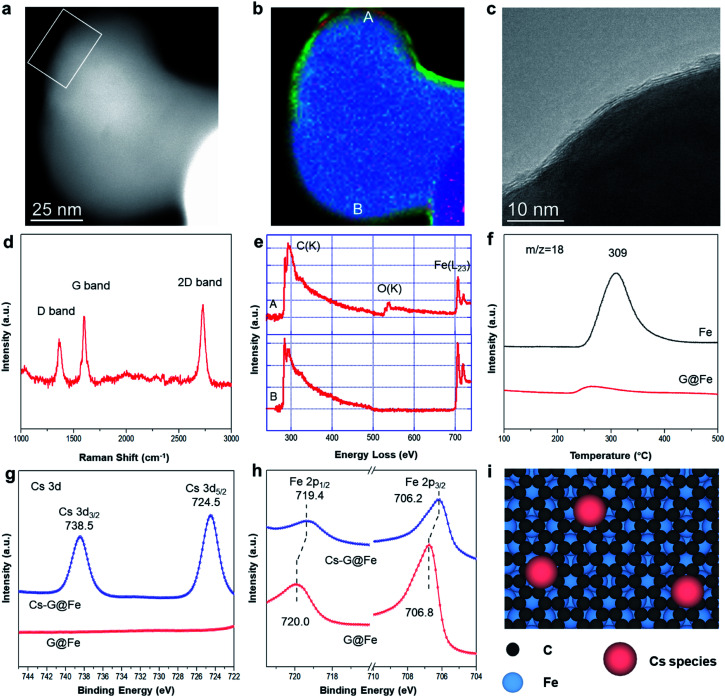
Characterization of the catalysts. STEM (a) and elemental mapping (b) images of G@Fe. Blue: Fe, green: C, red: O. (c) High resolution TEM image of the edge of G@Fe particle in white square of (a). (d) Raman spectra of G@Fe. (e) EELS spectra by scanning the regions of A and B in (b). (f) H_2_-TPSR profiles of Fe and G@Fe. Cs 3d (g) and C 1s (h) region of high energy resolution pseudo-*in situ* XPS of catalysts. (i) Proposed structure of Cs-G@Fe catalyst, the number of graphene layers could be 1–3.

The G@Fe catalyst was synthesized with a chemical vapor deposition method reported elsewhere,^22^ and details are described in Fig. S1.† Continuous graphene overlayers (1–3 layers) on the Fe surface can be clearly discerned by the high resolution TEM and the corresponding EELS elemental mapping analysis (Fig. 1a–c and S2†). The presence of the graphene overlayer is further confirmed by the *I*_2D_/*I*_G_ ratio and the width of 2D band^22^,^23^ of Raman spectrum (Fig. 1d and S3†). The D band in the spectrum reveals that the graphene layer has a certain amount of the defects^24^ which may act as the anchoring sites of metal dopants.^25^ In the EELS spectra of G@Fe (Fig. 1e), the peak structure in the carbon K-edge region indicates sp^2^ bonding.^26^ Note that, before being exposed to air for microscopy, as-synthesized sample was passivated by exposure to 1% O_2_/N_2_. If the Fe surface is not fully covered by the graphene overlayers, oxidation of Fe by O_2_ would be inevitable. From Fig. 1b, majority of the surface Fe on G@Fe is protected from oxygen oxidation by the graphene overlayers as evidenced by absence of oxygen (further confirmed by EELS spectrum b in Fig. 1e), except a few minor oxygen patches such as spot a in Fig. 1b which is also confirmed by EELS spectrum a in Fig. 1e. The nearly full coverage and protection of Fe surface by graphene overlayers were further confirmed by examining the Raman spectra and H_2_-TPSR profiles of the Fe and G@Fe samples after oxygen exposure (*i.e.*, air for Raman and 2% O_2_/He for H_2_-TPSR). For the Fe sample, formation of surface iron oxides after exposure to oxygen was confirmed by both Raman (Fig. S4†) and H_2_-TPSR (water formation peak at 309 °C in Fig. 1f due to the reduction of Fe oxides by H_2_). In contrast, both Raman (Fig. S4†) and H_2_-TPSR (Fig. 1f) characterization shows the absence of iron oxides after the G@Fe sample was exposed to oxygen. These results strongly suggest that graphene is able to protect the surface Fe from oxidation. The G@Fe sample can be further modified with alkali metal (*i.e.* Cs) for the selective C–O bond cleavage, as suggested by homogeneous catalysis for the selective hydrogenolysis of aromatic ethers,^19^,^20^ which will be discussed in the following sections.

The surface electronic properties were further studied with XPS after a pseudo *in situ* pretreatment with H_2_ at 300 °C (simulating the surface under HDO reaction). Over G@Fe, C 1s spectrum displays a peak at 284.8 eV (Fig. S5†), characteristic of the sp^2^ carbon of graphene on Fe.^22^ The C in iron carbide was reported to be located at ∼283.4 eV (ref. 27) that may overlap with the signal of graphene. The highly symmetric peak shape, however, suggests the amount of carbide, if any, is minimal on the catalyst surface. The C 1s peak is also used as a reference for the charge correction of other species. Fig. 1g shows the Cs 3d region of G@Fe and Cs-G@Fe. Upon adding Cs on G@Fe, the Cs 3d spectrum shows the binding energy of 724.5 eV for Cs 3d_5/2_ and 738.5 eV for 3d_3/2_.^28^,^29^ In Fe 2p spectrum of G@Fe (Fig. 1h), two peaks centered at 706.8 eV for 2p_3/2_ and 720.0 eV for 2p_1/2_ are observed, characteristic of metallic Fe.^30^ Notably, doping Cs on the surface shifts the Fe peaks to lower binding energy by 0.6 eV, suggesting that the Fe becomes more electron rich^31^ due to electron donation from Cs to Fe through the graphene overlayers. This charge transfer *via* electron tunneling has been widely demonstrated on metal covered with conductive graphene layers^32^ or even a thin layer of oxide insulator.^33^,^34^ Based on these results, the surface structure of Cs-G@Fe catalyst can be inferred as shown in Fig. 1i. Briefly, the graphene layer is deposited on Fe substrate,^27^,^35^ while the Cs species is anchored on graphene, which further modifies the electronic properties of Fe.

### Catalytic performances

**Fig. 2:**
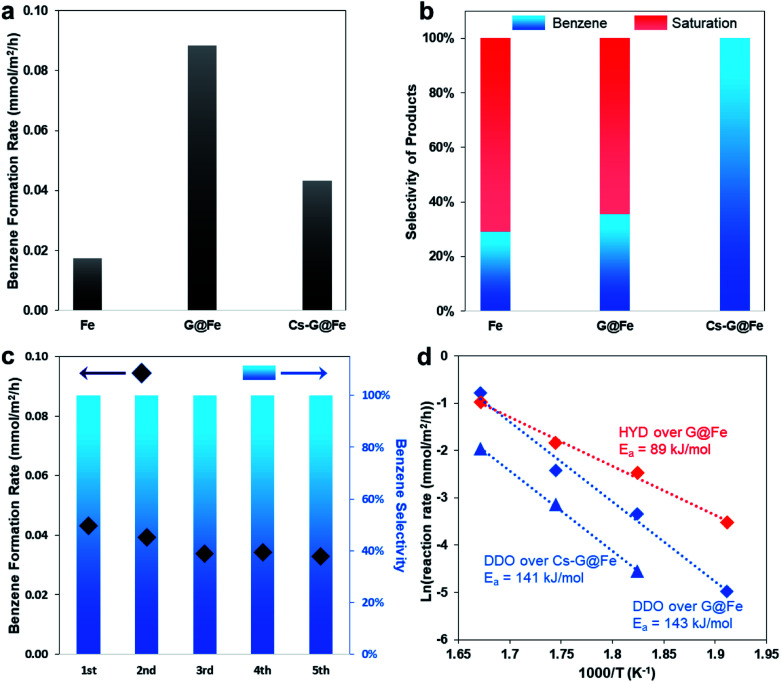
Catalytic performances in HDO of phenol. (a and b) Apparent benzene formation rate (a) and product selectivity (b) over different catalysts in a 4 h reaction. Reacting condition: 300 °C, 50 mL hexadecane as solvent, 1.6 MPa H_2_, 0.15 g catalyst, 0.6 g phenol, 4 h. (c) Stability test of Cs-G@Fe in HDO of phenol under same reacting condition, each cycle took 4 h. (d) Arrhenius plot for G@Fe and Cs-G@Fe. Reacting condition: 50 mL hexadecane, 1.6 MPa H_2_, 0.15 g catalyst, 0.6 g phenol. Apparent activity was obtained at conversions below 15%.

As previously mentioned, although several catalysts, including Fe-based ones, have been reported to selectively produce arenes in the gas-phase HDO of phenolics, aromatic ring-saturation reactions (*e.g.* producing cyclohexane and cyclohexanol in HDO of phenol) dominate on these catalysts under the liquid-phase conditions. Herein, using phenol as a probe molecule, we evaluated the bare Fe (BET surface area: 9.2 m^2^ g^–1^), G@Fe (BET surface area: 4.5 m^2^ g^–1^) and Cs-G@Fe (BET surface area: 4.5 m^2^ g^–1^) in liquid-phase HDO. As shown in Fig. 2a and b, the bare Fe catalyst showed both low activity and poor selectivity to benzene in a 4 hour reaction. G@Fe exhibited 4 times higher activity than the bare Fe. Separate time-on-stream tests showed drastic deactivation of the bare Fe catalyst, whereas the deactivation was significantly mitigated on the G@Fe catalyst (Fig. S6†). This observation, in alignment with the aforementioned characterization, suggests that graphene overlayers indeed protect the Fe from oxidation, leading to the stabilized activities on the metallic Fe and thus the higher apparent activity. Despite the improved apparent activity of G@Fe catalyst, benzene selectivity remained constantly low at ∼30%, suggesting that the nature of the active sites on G@Fe is same as that of bare Fe. In homogeneous catalysis, alkali metal additives have been used in the selective C–O bond cleavage of aromatic ethers with Ni-based complex catalysts.^19^,^20^ In this study, doping Cs on G@Fe significantly increased the benzene selectivity to 100% (Fig. 2a and b), likely due to the inhibited tautomerization. Note that alkali metals have been used as additives on other catalysts for selective hydrogenation of nitroarenes^36^ or promotion to aromatic ring saturation.^37^ Different from those reported ones, directly doping alkali metal on the Fe without graphene overlayers is not able to enhance the selectivity, instead the catalyst was almost completely deactivated. It suggests that the graphene overlayers, other than prevent Fe oxidation, must play another pivotal role to prevent the poison of surface Fe *via* a mediated Fe–alkali metal interaction, the reason of which remains unclear and subjects to further studies. Regardless, we first applied a similar approach used in homogeneous catalysis to the heterogeneous analogues and achieved the selective C–O bond cleavage of phenolics in HDO. It should be mentioned that the addition of alkali metal resulted in the decrease of apparent rate for benzene production as shown in Fig. 2a. This decrease can be expected since tautomerization reaction pathway also contributed to benzene formation (Fig. S7†), and the Cs addition completely shut down the tautomerization pathway. The stability of the representative Cs-G@Fe was also studied and results are shown in Fig. 2c. Although a slight decrease in reactivity was observed after the 1^st^ cycle, the catalyst remained relatively stable afterwards for 4 additional testing cycles. More importantly, the benzene selectivity remained at 100% in all the tested cycles. It should also be mentioned that, due to the magnetic nature of the catalyst, the catalysts can be readily separated from the liquid phase by applying a magnetic field as demonstrated in the cyclic stability tests (Fig. S8†). The spent Cs-G@Fe after the HDO reaction was characterized with Raman spectroscopy and TEM (Fig. S9†). The 2D peak in Raman spectrum indicated the structure of carbon is still graphitic. TEM image showed the graphene overlayer was still intact and attached to the catalyst surface. These results indicate the catalyst surface structure remained the same as the one shown in Fig. 1i after the HDO reaction.

It has been well known, Fe single atom could be formed and stabilized on either defect sites of graphene^38^ or N doped graphene.^39^,^40^ This Fe species may serve as the active sites for DDO. From Raman spectra (Fig. 1d), however, the defect sites on G@Fe is minimum. In addition, although N_2_ gas was used during the synthesis of G@Fe, no N signal can be detected at ∼400 eV in the EELS (Fig. 1e) and XPS (Fig. S10†). It suggests that Fe single atom, if any, should be negligible on the G@Fe catalyst. Here, we postulate that the actives sites are likely Fe covered with several graphene overlayers through which electron tunneling takes place.^32^ A similar observation has been reported in electrocatalysis where graphene covered Ni–Co alloy^3^ and Fe_3_C^41^ allow electrons moving through these graphene overlayers while protecting Ni–Co and Fe_3_C in acid electrolytes.

The binding energy of Fe is lowered by 0.6 eV after adding Cs, as shown in Fig. 1h. This implies that the addition of Cs leads to the charge transfer and, consequently, the tuned surface electronic properties of the graphene-protected Fe and the catalyst functionalities.^42^ The change of electronic properties has been shown to influence the activation energy (*E*_a_) of a reaction.^43^ Therefore, separate kinetic analysis was done to compare the apparent *E*_a_ of the reactions on the G@Fe and Cs-G@Fe (Fig. 2d). The reaction pathways are categorized into two types: direct deoxygenation (DDO) producing benzene and hydrogenation followed by deoxygenation (HYD) producing ring-saturated compounds (Fig. S11†). Over G@Fe, both pathways exist. The apparent *E*_a_ of HYD (89 kJ mol^–1^) is much lower than that of DDO (143 kJ mol^–1^). This is consistent with the higher bond dissociation energy of C_aromatic_–O^44^,^45^ and the results from previously reported kinetic studies.^46^ Over the Cs-G@Fe, the ring saturation was inhibited (*i.e.* Cs significantly increased *E*_a_ for ring saturation) and the apparent *E*_a_ of direct C–O bond cleavage is 141 kJ mol^–1^ which is comparable with that of DDO on G@Fe (143 kJ mol^–1^). This implies that Cs may not be directly involved in C–O bond cleavage, but instead, is primarily responsible for inhibition of the ring-saturation pathway.

### Possible reaction mechanisms

**Fig. 3:**
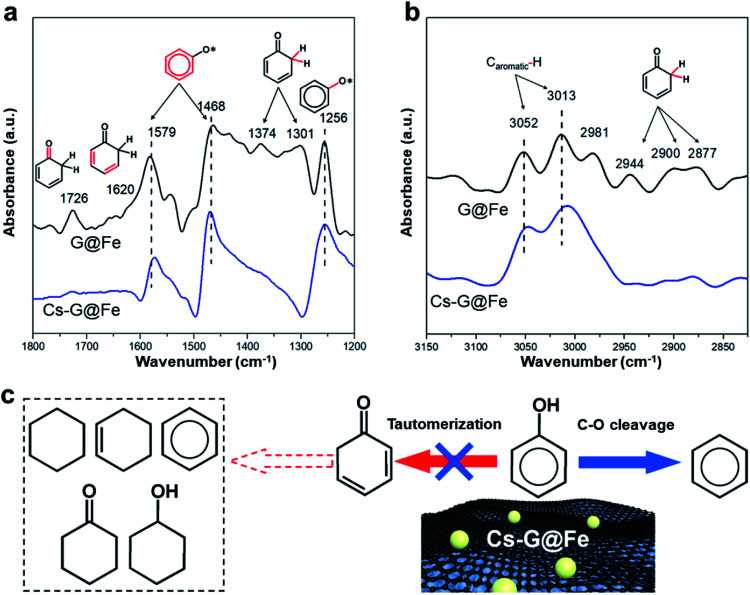
Spectroscopic studies of reaction mechanisms. (a and b) *In situ* ATR-FTIR spectra at different regions for phenol adsorption on G@Fe and Cs-G@Fe. The bond vibration attributed to each specific peak is highlighted in red. (c) Proposed reaction mechanisms for the selective HDO over Cs-G@Fe.

Previous results^16^ have shown that, while direct C–O bond cleavage selectively produces arenes, tautomerization mainly contributes to the ring saturation on the Fe-based catalysts in liquid-phase reactions (Fig. S7† and notation). Based on the fact that the *E*_a_ for DDO remains the same after Cs addition, it is hypothesized that the addition of Cs largely inhibits the tautomerization reaction pathway but has a minimal effect on the C–O bond cleavage. To further verify this hypothesis, *in situ* ATR-FTIR was employed to investigate the phenol adsorption over G@Fe and Cs-G@Fe. As shown in Fig. 3a, both spectra of G@Fe and Cs-G@Fe display peaks at 1579, 1468 cm^–1^ (stretching vibrations of the aromatic ring)^47^ and 1256 cm^–1^ (stretching vibration of the C–O bond),^48^ an indication of surface adsorbed phenol species. Most importantly, in contrast to the Cs-doped surface, G@Fe exhibits other peaks or shoulder at 1726, 1620, 1374, 1301 cm^–1^, which can be assigned to stretching vibration of C=O bond,^49^ stretching of aliphatic C=C bond,^49^ wagging and twisting vibrations of CH_2_ (ref. 48) of surface cyclohexadienone, respectively. This observation suggests that cyclohexadienone intermediate is indeed formed from the tautomerization of phenol^50^ in the absence of Cs, which is further supported by C–H stretching region as shown in Fig. 3b. Both spectra display peaks at ∼3052 and ∼3013 cm^–1^, which are attributed to C–H vibrations in the aromatic ring.^51^,^52^ The spectrum of G@Fe also shows other peaks in 2944–2877 cm^–1^, characteristic of stretching of C_sp^3^_–H in cyclohexadienone species.^50^ The above results are aligned with other infrared spectroscopic studies that also observed the keto intermediates from tautomerization in the HDO of phenol.^50^,^53^ The fact that no tautomerization intermediates were observed on Cs doped G@Fe confirms that surface Cs species is indeed able to inhibit tautomerization and thus leads to selective C–O bond cleavage of phenol, as elucidated in Fig. 3c. One possible interpretation for this selective catalysis is there may be two types of active sites on G@Fe (*i.e.*, one for tautomerization and another for selective C–O bond cleavage), and depositing Cs may selectively block the one for tautomerization with the other one being exposed to phenol for C–O cleavage. To test this hypothesis, we further compared the performances of other alkali metal doped catalysts with same molar loading (Fig. S12†). Given the slight difference of ionic sizes between K and Na,^54^ if the alkali metals serve as the block site, a similar catalytic performance in terms of benzene selectivity can be expected over K-G@Fe and Na-G@Fe. The fact that a significant different benzene selectivity was observed on K-G@Fe (95%) and on Na-G@Fe (44%) suggest that the alkali metal may not be a site blocker. In contrast, the inhibition of tautomerization (*i.e.*, benzene selectivity) is well correlated with the capability of electron donation of alkali metal^21^ to the Fe (Fig. S12†). Therefore, though the electronic structure and the specific roles of Cs-G@Fe remain unclear, we propose the charge transfer from Cs to the active site (*i.e.* graphene-covered Fe) may play pivotal roles to inhibit the functionality for catalyzing tautomerization of phenol. While allowing the electron tunneling, graphene overlayers may mediate the interaction between Fe and alkali metal/substrate oxygen to prevent Fe from deactivation.

## Conclusions

Our results present an approach to protect Fe with graphene and further tune the G@Fe catalyst with alkali metal for selective hydrogenolysis of C–O bond in HDO of phenol. Graphene overlayers on Fe protect Fe from oxidation while, at the same time, maintaining the nature of Fe as confirmed by its catalytic activity resembling that of bare Fe. More importantly, analogous with homogeneous catalysis, alkali metals such as Cs could be added to tailor the surface electronic properties of G@Fe, leading to the selective inhibition of tautomerization and thus exclusive C–O bond cleavage in the heterogeneously catalyzed HDO of phenolics (*i.e.*, phenol). To our best knowledge, this is the first sulfur-free inexpensive catalyst reported for exclusive hydrogenolysis of the C–O bond in phenolics under liquid-phase conditions. Moreover, the catalyst also offers other beneficial properties, *i.e.* magnetic material for a facile separation from reaction slurry, making it a promising catalyst for liquid-phase reactions. This work could also lead to a general methodology for rational design of heterogeneous catalysts for selective HDO of oxygenates.

## Conflicts of interest

There are no conflicts to declare.

## Supplementary Material

Supplementary information
