# Supplementary material for: Surface engineering of earth-abundant Fe catalysts for selective hydrodeoxygenation of phenolics in liquid phase
Source: Chem Sci. 2020 May 18;11(23):5874–80. doi: 10.1039/d0sc00983k (PMC7448371; doi:10.1039/d0sc00983k)
Supplement: Supplementary file 1 [file SC-011-D0SC00983K-s001.pdf]

Electronic Supplementary Material (ESI) for Chemical Science.
This journal is © The Royal Society of Chemistry 2020

Supporting Information

Surface Engineering of Earth-abundant Fe Catalysts for Selective Hydrodeoxygenation of Phenolics in Liquid Phase

Jianghao Zhang,[a] Junming Sun,*[a] Libor Kovarik,[b] Mark H. Engelhard,[b] Lei Du,[c] Berlin Sudduth,[a] Houqian Li,[a] Yong Wang*[a, b]

a *The Gene & Linda Voiland School of Chemical Engineering and Bioengineering, Washington State University, Pullman, WA 99164, USA.*
E-mail: junming.sun@wsu.edu; yong.wang@pnnl.gov

b *Institute for Integrated Catalysis and Environmental Molecular Sciences Laboratory, Pacific Northwest National Laboratory, Richland, WA 99352, USA.*

c *School of Chemistry and Chemical Engineering, Harbin Institute of Technology, Harbin 150001, CN.*

Table of Contents

Experimental Procedures 3
Results and Discussions 4
Fig. S1 A scheme of G@Fe and Cs-G@Fe synthesis. 4
Fig. S2 TEM images (a-c) and EELS spectrum (d) of G@Fe. 5
Fig. S3 Raman spectra taken at different spots on G@Fe. 6
Fig. S4 (a) A comparison about the Raman spectra of Fe and G@Fe. (b) Spectra of different spots on G@Fe. 7
Fig. S5 XPS spectra for C 1s line of G@Fe and Cs-G@Fe. 7
Fig. S6 Benzene formation rate over Fe and G@Fe in the time-on-stream phenol HDO tests. Inset: the extrapolation of benzene formation rate to 0 h. Reaction conditions: 300 °C, 1.6 MPa H_2, 0.15g catalyst, 0.6 g phenol, conversion < 15% 8
Fig. S7 The three reported primary reaction pathways in HDO of phenol. 9
Fig. S8 Magnetic separation of liquid and catalysts after reaction: before (a) and after (b) imposing magnetic field 9
Fig. S9 TEM (a) and Raman spectrum (b) of spent Cs-G@Fe. 10
Fig. S10 XPS spectrum of G@Fe. 10
Fig. S11 Two general reaction pathways in HDO of phenol. 11
Fig. S12 Reported effective ionic radius of Na, K, Cs normalized to the value for Cs (a). Normalized change of computed net charge of Fe modified with different alkali metals (b). Product selectivity (c), normalized benzene formation rate (d) and ring saturation rate (e) over different catalysts in HDO of phenol. Reacting condition: 50 mL hexadecane, 1.6 MPa H_2, 0.15 g catalyst, 0.6 g phenol. 11
References 12

Experimental Procedures

Preparation of catalysts. The Fe_2O_3 was prepared with a precipitation method. Typically, $(NH_4)_2CO_3$ (Sigma-Aldrich, ≥99.999%, 1.5M) solution was added dropwise to a solution of $Fe(NO_3)_3$ (Sigma-Aldrich, ≥99.99% or ≥98%, 3M) under stirring, forming a dark crimson slurry. The precipitate was washed with Milli-Q water via filtration until the pH reached 8, and then transferred into a convection oven for drying at 80 °C overnight. The obtained solid sample was then crushed and sieved to <100 mesh, followed by a calcination at 400 °C for 5 hours to obtain the Fe_2O_3.

G@Fe was prepared by chemical vapor deposition method using CH_4 as the carbon source. After reduction of Fe_2O_3 at 350 °C for 12h in alumina boat placed in a quartz tube horizontally inserted in a tube furnace, the reduced Fe was cooled down to room temperature where the gas was switched to 5 kPa CH_4 balanced with N_2 mixture (50 mL/min) for a 1 h purge. Then the temperature was ramped to 700 °C at a rate of 50 °C /min and held for 2 min, followed by simultaneously purging with N_2 (100 mL/min) and cooling down to room temperature rapidly. Finally, the catalyst sample was passivated with 1 vol.% O_2/N_2 (50 mL/min) for 2 h. To obtain the Cs-G@Fe, Cs_2CO_3 powder (Sigma-Aldrich, 99.9%, metal basis) was dissolved by a calculated amount of Milli-Q water such that the concentration and volume of the precursor solution matched with pore volume of the Fe as well as the Cs loading (0.85 wt.%) of the obtained catalysts. The transparent solution was then added dropwise to G@Fe powder, followed by stirring the support/solution mixture for approximately 10 min to reach an even dispersion of solution over the surface. The mixture was then transferred into an alumina boat placed in a quartz tube horizontally inserted in a tube furnace with flowing UHP N_2 (50 mL/min). After purging for 1 h, the sample temperature was increased to 80 °C (5 °C/min) and held for 6 h, and then to 400 °C and held for another 2h. After cooling to ambient temperature in flowing UHP N_2 (50 mL/min), the sample was passivated by flowing 1 vol.% O_2/N_2 (50 mL/min) for 2 h.

Characterizations

Scanning Transmission Electron Microscopy (STEM) was conducted on a FEI Titan 80–300 operated at 300 kV. The images were obtained with a High Angle Annular Dark Field Detector (HAADF) in Scanning Transmission Electron mode. The semi-convergence angle was set to 17.8 mrad, and the inner collection angle was set above 54 mrad. Compositional analysis was performed with a JEOL ARM 200 operated at 200 kV. The microscope houses a high-collection angle Silicon Drift Detector SDD ($100mm^2$). After passivation (flowing $1\%O_2/N_2$ and air, successively) of the synthesized G@Fe, it was stored in a vial filled with N_2 before the STEM imaging. In the preparation of sample, a dry powder was dispersed on a lacey-carbon coated 200 mesh Cu grids.

Transmission electron microscopy (TEM) analysis of the catalysts was performed using a FEI Technai G2 20 Twin operated at 200 kV and equipped with a 4k Eagle CCD camera. To prepare the samples for TEM test, several milligrams of sample was suspended into ethanol with a sonicator, then a drop of the nanoparticle suspension was dispensed onto a 3-mm copper grid with lacey carbon films. Excess ethanol was removed by an absorbent paper, and the sample was dried at room temperature for 6 hours.

To evaluate the efficiency of magnetic separation of catalyst, a magnetic stirring bar was placed beside the mixture of catalyst and liquid containing the solvent, reactants and products after a 4-h reaction. The picture was taken after 10 s of separation.

Raman spectra were collected on a Horiba LabRAM HR Raman/FTIR microscope equipped with a 532 nm (Ventus LP 532) laser source and Synapse Charge Coupled Device detector (CCD). In a typical measurement, the sample was loaded in a Linkam CCR1000 *in situ* cell and purged with N_2 (50 ml/min) for 10 min. After calibration using the silica reference, the spectrum of the sample was taken at room temperature. H_2-TPSR was carried out on Chemisorption Analyzer (Micromeritics AutoChem 2920) equipped with a quadrupole mass spectrometer (Omnistar gas analyzer GSD 301). Specifically, 0.1 g sample was loaded into a quartz tube and pretreated with 10 vol.% H_2/Ar at 300 °C for 1 h. Then the temperature dropped to 0 °C followed by 0.5 min 2% O_2/He passivation with flow rate of 100 mL/min. After switching to 10 vol.% H_2/Ar and waiting for 20min, the temperature was ramped to 800 °C (10 °C/min).

Pseudo-*in situ* XPS measurements were performed after pseudo *in-situ* processing under 100 SCCM UHP H_2 at 300°C for 1 hr. Sample were cooled in H_2 to room temperature, the H_2 was quickly pumped to UHV and transferred in UHV into the XPS spectrometer for

analysis. XPS measurements were performed with a Physical Electronics Quantera Scanning X-ray Microprobe. This system uses a focused monochromatic Al Kα X-ray (1486.7 eV) source for excitation and a spherical section analyzer. The instrument has a 32 element multichannel detection system. The X-ray beam is incident normal to the sample and the photoelectron detector is at 45° off-normal. High energy resolution spectra were collected using a pass-energy of 69.0 eV with a step size of 0.125 eV. For the Ag 3d5/2 line, these conditions produced a FWHM of 0.92 eV ± 0.05 eV. The binding energy (BE) scale is calibrated using the Cu 2p3/2 feature at 932.62 ± 0.05 eV and Au 4f7/2 at 83.96 ± 0.05 eV. Low energy electrons at ~1 eV, 20μA and low energy Ar+ ions were used to minimize variable charging effect. Charge referencing was made using the binding energy for C 1s of graphene at 284.8 eV[1].

In situ ATR-FTIR was employed to study the tautomerization of adsorbed phenol on the catalysts. The measurements were performed using Bruker Tenser Ⅱ spectrometer and a custom-designed ATR cell as described elsewhere[2]. Briefly, the as-prepared catalysts were ground into very fine powders. Then, samples were sonicated in hexane to form suspension that was deposited on the ZnSe Internal Reflection Element by dip coating. The thin layer of catalyst coating was then reduced in 10%H_2/He (40 mL/min) at 300 °C for 1h. After that, the temperature was cooled down to ambient followed by N_2 purging for 1h. After acquiring a background spectrum, 10 vol.% CO/He was introduced to the catalyst. The spectra were then recorded (64 scans at a resolution of 4 cm^{-1}).

Evaluation of catalytic performance

The HDO reaction of phenol was carried out in a stainless steel Parr reactor (Series 4560, 300 mL) equipped with a glass liner. In a typical measurement, 0.15 g catalyst, 0.6 g phenol (Sigma-Aldrich, ≥ 99%) and 50 mL hexadecane (Sigma-Aldrich, 99%) were loaded into the reactor. After a leak test and 3 times of purging with 4MPa H_2, the temperature was ramped to 300 °C (15 °C/min) followed by pressurizing with H_2 to 1.6 MPa where the stirring (800 rpm) of the mixed slurry solution and reaction started. After the reaction is completed, the products were analyzed on a gas chromatograph (GC, Agilent 7890A) equipped with a DB-FFAP column (30m, 0.32mm, 0.25μm) and flame ionization detector (FID), as well as a GC-MS (Shimadzu, GC-MS-QP2020) equipped with a HP-5 column (30m, 0.32mm, 0.25μm) connected to a FID. The benzene formation rate (measured with conversions below 15%) and selectivity were defined as follows: benzene formation rate [mmol/m^2/h] = mole of produced benzene/(amount of catalyst × BET surface area)/reacting time; selectivity [%] = (moles of carbon in the specific product/moles of carbon in all product) × 100%. The carbon balance was above 92%.

Results and Discussions

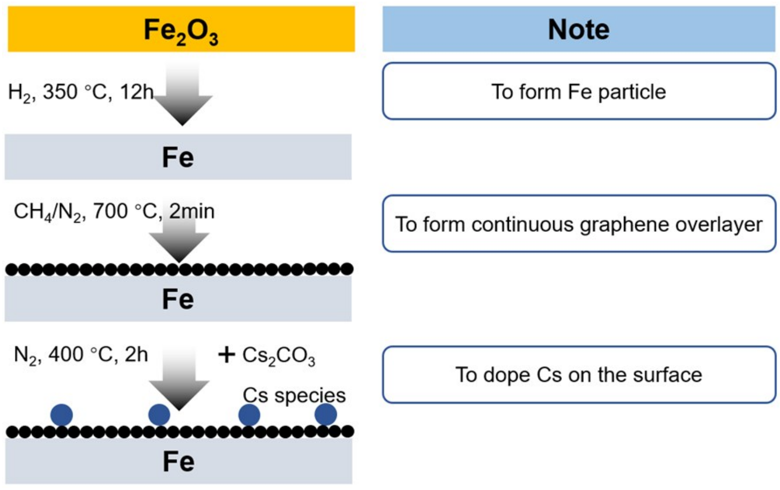

Fig. S1 A scheme of G@Fe and Cs-G@Fe synthesis.

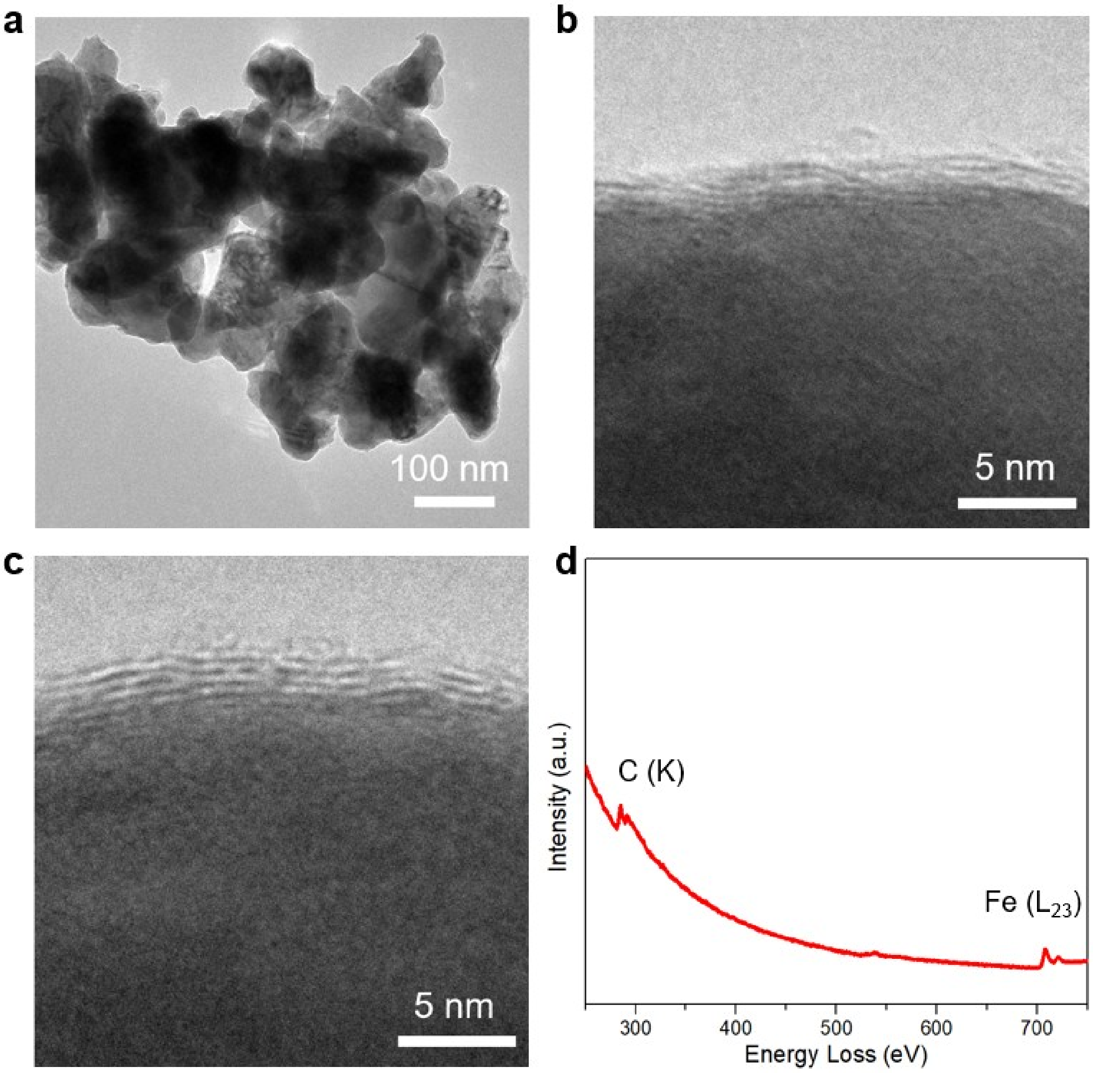

Fig. S2 TEM images (a-c) and EELS spectrum (d) of G@Fe.

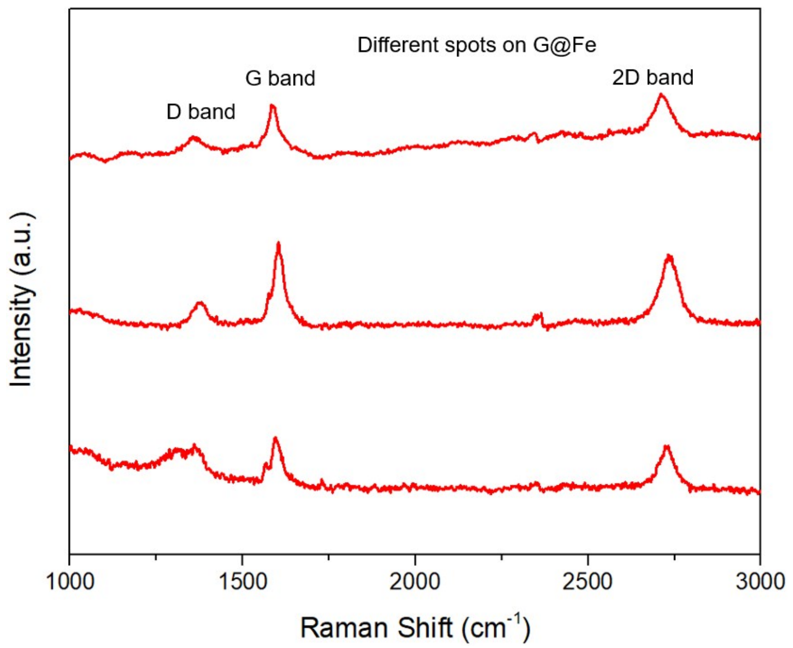

Fig. S3 Raman spectra taken at different spots on G@Fe. 2D band is the most prominent feature in the Raman spectrum of graphene[1]. In each Raman spectrum at several different spots of G@Fe sample, the 2D band is observed, suggesting the graphene layer is covering most area of the Fe substrate. While the smaller D band (relative to the G and 2D bands) in the spectra indicates that the graphene layer has a little amount of the defects[3] which may act as the anchoring sites of metal dopants[4].

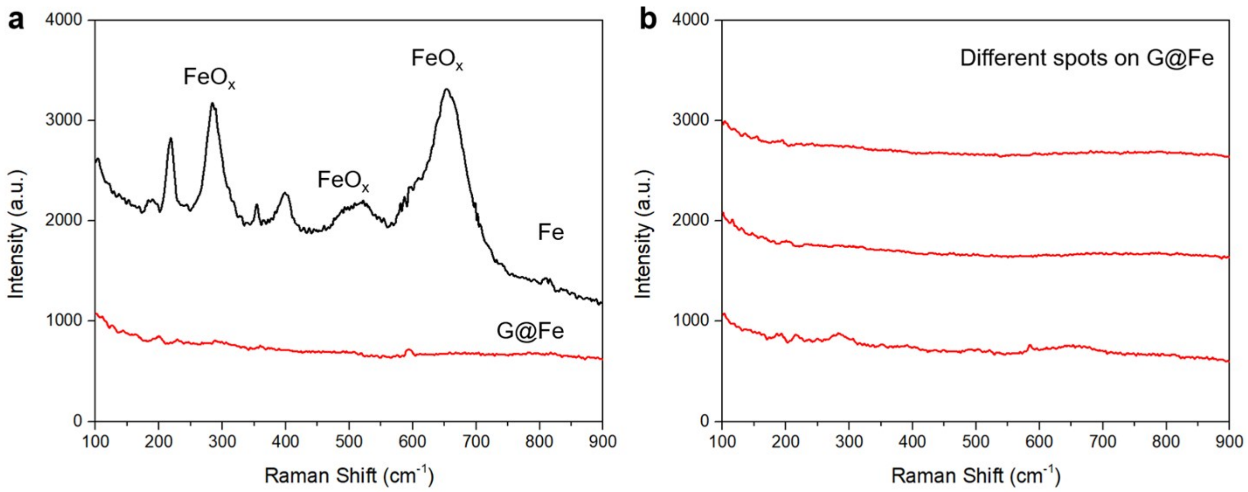

Fig. S4 (a) A comparison about the Raman spectra of Fe and G@Fe. (b) Spectra of different spots on G@Fe. After exposure to O_2, there was clear surface oxidation of the bare Fe sample, as detected by the characteristic peaks at 219, 284, 400, and 654 cm^{-1} [5]. Due to the protection of the graphene overlayer, the Fe in G@Fe is not oxidized after exposure to O_2.

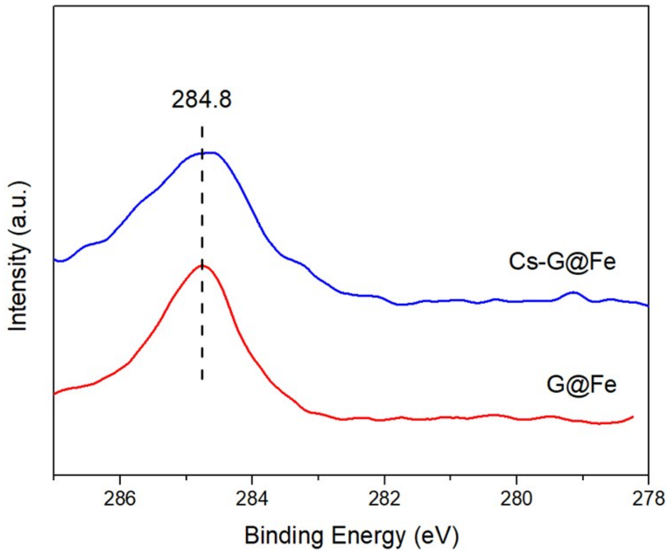

Fig. S5 XPS spectra for C 1s line of G@Fe and Cs-G@Fe.

The charge correction was made with C 1s at 284.8 eV for graphene on Fe[1]. This is corroborated with the Fe 2p3/2 line at 706.8 eV[6]. The C in iron carbide was reported to be located at ~283.4 eV[7] that may overlap with the signal of graphene. The highly symmetric peak shape suggests the amount of carbide, if any, is minimal on the catalyst surface.

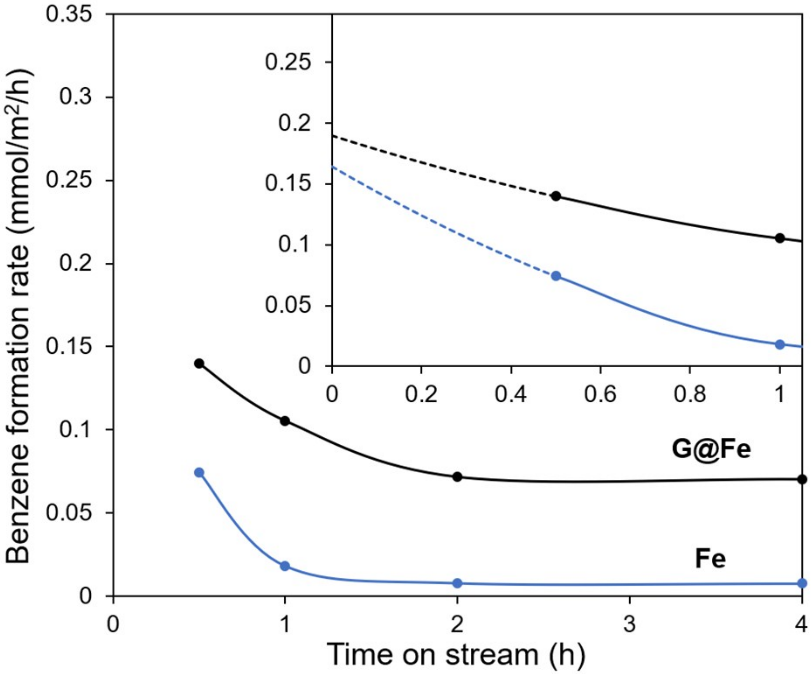

Fig. S6 Benzene formation rate over Fe and G@Fe in the time-on-stream phenol HDO tests. Inset: the extrapolation of benzene formation rate to 0 h. Reaction conditions: 300 °C, 1.6 MPa H_2, 0.15g catalyst, 0.6 g phenol, conversion < 15%. Bare Fe drastically deactivated during the HDO reaction, while the graphene overlayer significantly mitigated deactivation of G@Fe. Extrapolating the reactivity curve of Fe and G@Fe in time-on-stream tests shows the two catalysts have comparable initial activity, which suggests the high apparent reactivity of G@Fe is mainly due to mitigated deactivation by the graphene overlayer.

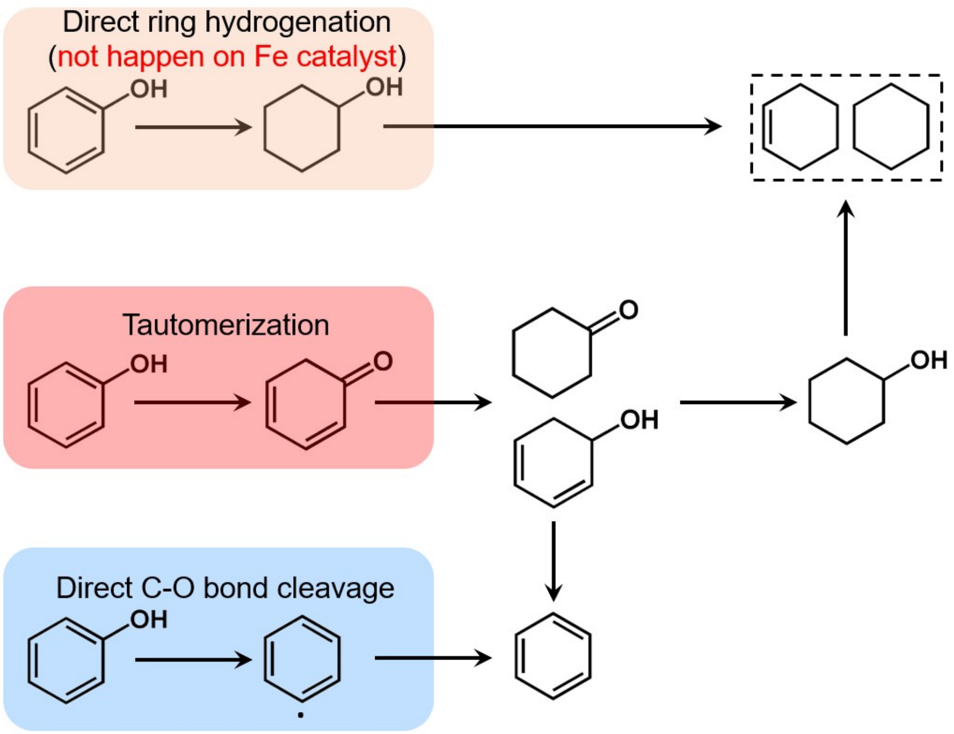

Fig. S7 The three reported primary reaction pathways in HDO of phenol.

In general, three primary reaction pathways have been proposed in the HDO of phenols: direct aromatic ring hydrogenation, keto-enol tautomerization and direct C-O bond cleavage[8]. Our previous study has shown that the direct ring hydrogenation does not occur over the Fe-based catalyst[9] in liquid-phase HDO. Moreover, at high H_2 partial pressure, direct ring saturation pathway is not able to produce benzene because the dehydrogenation of cyclohexene to benzene is thermodynamically unfavorable. Also, high H_2 pressure can enhance the hydrogenation of intermediates in the tautomerization pathway, resulting in decreased benzene selectivity. On the other hand, direct C-O bond cleavage still forms benzene as a primary product. Our recent work investigated the role of tautomerization in hydrodeoxygenation of the lignin-derived phenolics by employing two representative modeling compounds: phenol (a molecule that is keto-enol tautomeric) and diphenyl ether (a molecule that does not allow ketol-enol tautomerization). It was demonstrated that over Fe-based catalysts, the tautomerization pathway is the major route for ring saturation in the liquid-phase HDO of phenol at high H_2 pressure[9].

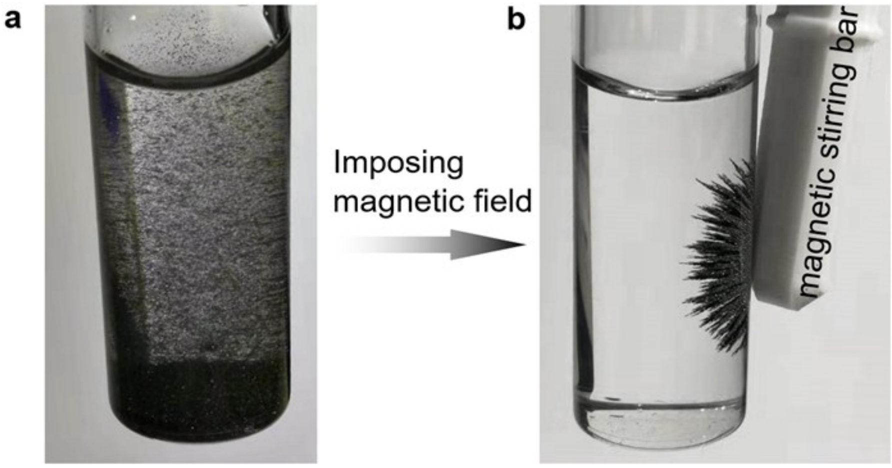

Fig. S8 Magnetic separation of liquid and catalysts after reaction: before (a) and after (b) imposing magnetic field. The Cs-G@Fe can be readily separated from the reaction mixture using an external magnetic field. This property benefits the application in liquid-phase reaction[10].

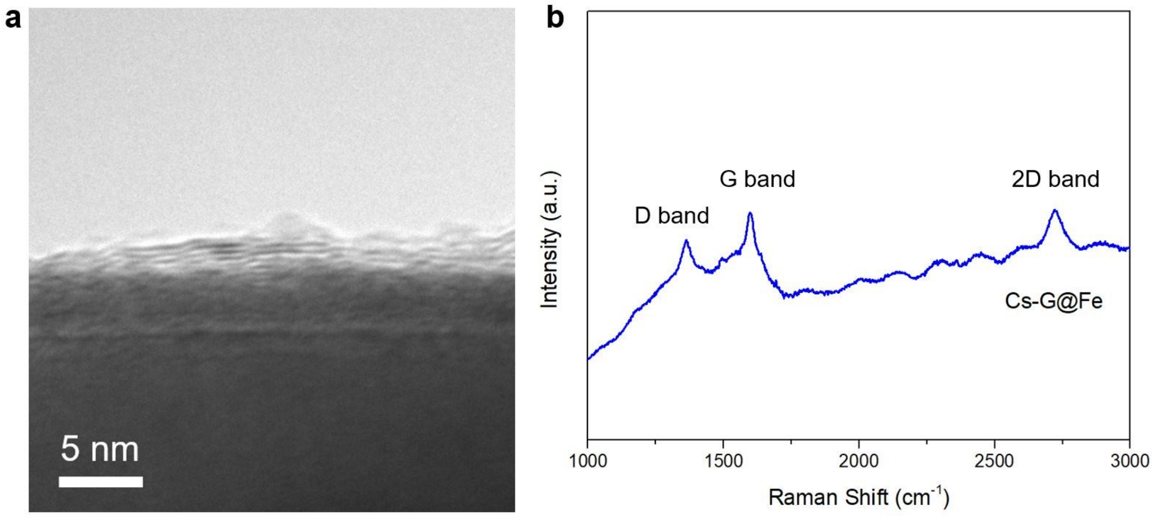

Fig. S9 TEM (a) and Raman spectrum (b) of spent Cs-G@Fe.

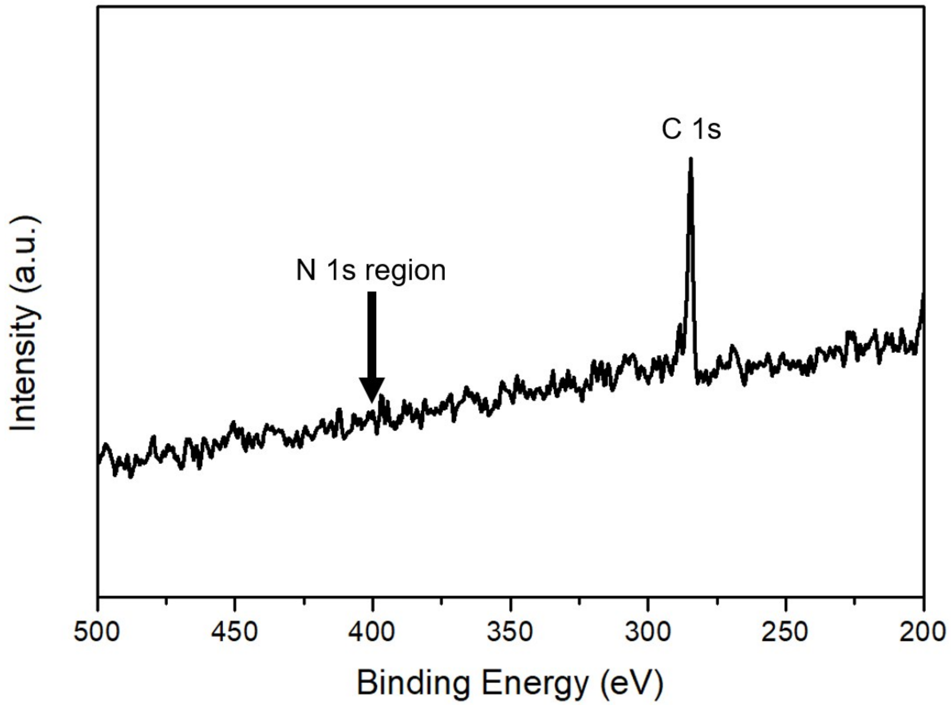

Fig. S10 XPS spectrum of G@Fe. Note that N 1s line is located at ~400 eV[11]. The absence of N peak indicates the N content is below the detection limit of XPS.

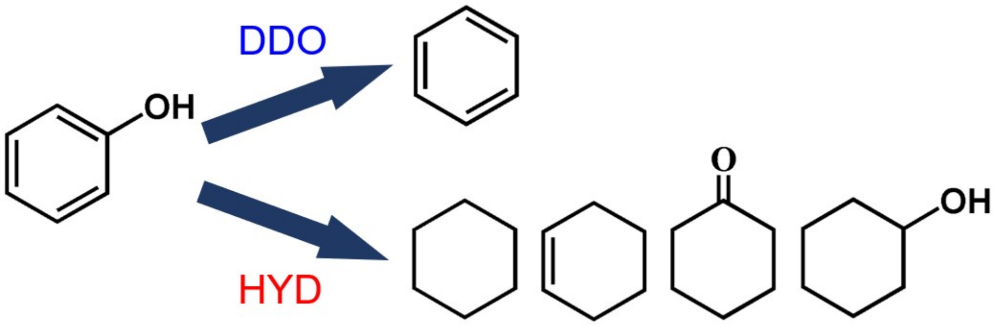

Fig. S11 Two general reaction pathways in HDO of phenol.

The reaction pathways are categorized into two types: direct deoxygenation (DDO) producing benzene and hydrogenation followed by deoxygenation (HYD) producing ring-saturated compounds[12].

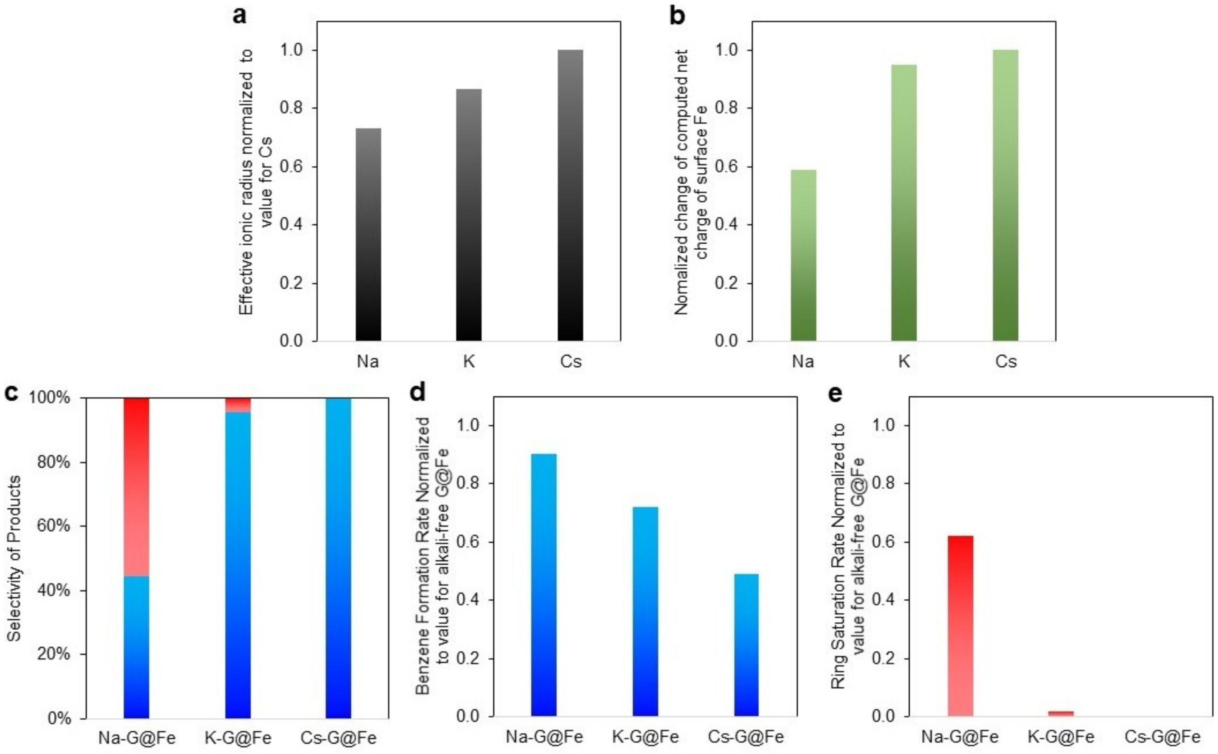

Fig. S12 Reported effective ionic radius of Na, K, Cs normalized to the value for Cs (a). Normalized change of computed net charge of Fe modified with different alkali metals (b). Product selectivity (c), normalized benzene formation rate (d) and ring saturation rate (e) over different catalysts in HDO of phenol. Reacting condition: 50 mL hexadecane, 1.6 MPa H_2, 0.15 g catalyst, 0.6 g phenol.

Fig. S12a shows the normalized effective ionic radius of Na, K, Cs (Cs is set as 1)[13]. Fig. S12b shows the normalized change of net charge of Fe led by doping different alkali metals, in comparison with alkali-metal-free sample[14]. We impregnated G@Fe with alkali metals of the same molar amount (0.15 wt.% for Na, 0.25 wt.% for K and 0.85 wt.% for Cs) and evaluated the catalytic performances under same reacting condition. It should be noted that though the difference of ionic size of K (0.87) and Na (0.73) are small, if the alkali metals play the role to block the active site for tautomerization, we should expect the similar benzene selectivity over K-G@Fe and Na-G@Fe, which is not consistent with Figure c, d and e that K shows a much stronger inhibition to ring saturation than Na. Instead, the change of net charge of Fe (Fig. 12b) is correlated well with catalytic performance. Therefore, it is inferred that the increased DDO selectivity should not be ascribed to selective blocking effect to site for tautomerization. Another model is proposed, there is only one type of active site which has two functionalities: catalyzing tautomerization and direct C-O cleavage. The role of alkali is modifying the electronic structure of G@Fe to eliminate the functionality for tautomerization.

References

[1] Y. Xue, B. Wu, Y. Guo, L. Huang, L. Jiang, J. Chen, D. Geng, Y. Liu, W. Hu, G. Yu, *Nano Res.* **2011**, *4*, 1208-1214.
[2] Z. H. Wei, A. M. Karim, Y. Li, D. L. King, Y. Wang, *J. Catal.* **2015**, *322*, 49-59.
[3] Z. H. Ni, W. Chen, X. F. Fan, J. L. Kuo, T. Yu, A. T. S. Wee, Z. X. Shen, *Phys. Rev. B* **2008**, *77*.
[4] O. Cretu, A. V. Krasheninnikov, J. A. Rodriguez-Manzo, L. Sun, R. M. Nieminen, F. Banhart, *Phys. Rev. Lett.* **2010**, *105*, 196102.
[5] aD. Bersani, P. P. Lottici, A. Montenero, *J. Raman Spectrosc.* **1999**, *30*, 355-360; bD. L. A. de Faria, S. Venâncio Silva, M. T. de Oliveira, *J. Raman Spectrosc.* **1997**, *28*, 873-878.
[6] A. K. Opitz, A. Nenning, C. Rameshan, R. Rameshan, R. Blume, M. Havecker, A. Knop-Gericke, G. Rupprechter, J. Fleig, B. Klotzer, *Angew. Chem. Int. Ed.* **2015**, *54*, 2628-2632.
[7] aN. A. Vinogradov, A. A. Zakharov, V. Kocevski, J. Rusz, K. A. Simonov, O. Eriksson, A. Mikkelsen, E. Lundgren, A. S. Vinogradov, N. Martensson, A. B. Preobrajenski, *Phys. Rev. Lett.* **2012**, *109*, 026101; bJ. P. Reymond, P. Meriaudeau, S. J. Teichner, *J. Catal.* **1982**, *75*, 39-48.
[8] G. H. Gu, C. A. Mullen, A. A. Boateng, D. G. Vlachos, *ACS Catal.* **2016**, *6*, 3047-3055.
[9] J. Zhang, J. Sun, B. Sudduth, X. Pereira Hernandez, Y. Wang, *Catal. Today* **2020**, *339*, 305-311.
[10] aG. Zhu, K. Wu, L. Tan, W. Wang, Y. Huang, D. Liu, Y. Yang, *ACS Sustain. Chem. Eng.* **2018**, *6*, 10078-10086; bZ. Zhang, J. Zhen, B. Liu, K. Lv, K. Deng, *Green Chem.* **2015**, *17*, 1308-1317.
[11] W. Liu, L. Zhang, X. Liu, X. Liu, X. Yang, S. Miao, W. Wang, A. Wang, T. Zhang, *J. Am. Chem. Soc.* **2017**, *139*, 10790-10798.
[12] G. Zhu, W. Wang, K. Wu, S. Tan, L. Tan, Y. Yang, *Ind. Eng. Chem. Res.* **2016**, *55*, 12173-12182.
[13] R. D. Shannon, *Acta Crystallographica Section A* **1976**, *32*, 751-767.
[14] T. Yang, X.-D. Wen, Y.-W. Li, J. Wang, H. Jiao, *Surf. Sci.* **2009**, *603*, 78-83.
